# Trends in hospital mortality of patients with status epilepticus in the ICU before and during the COVID-19 pandemic

**DOI:** 10.1097/MD.0000000000042219

**Published:** 2025-04-25

**Authors:** Lavi Oud, John Garza

**Affiliations:** a Department of Internal Medicine, Division of Pulmonary and Critical Care Medicine, Texas Tech University Health Sciences Center at the Permian Basin, Odessa, TX; b Department of Pediatrics, Texas Tech University Health Sciences Center at the Permian Basin, Odessa, TX.

**Keywords:** coronavirus disease 2019, intensive care unit, mortality, status epilepticus

## Abstract

The evidence on the temporal trends of short-term mortality among critically ill patients with status epilepticus (SE) is limited and relatively dated, with data lacking on the prognostic impact of the coronavirus disease 2019 (COVID-19) pandemic in these patients. We used statewide data to examine intensive care unit (ICU) admissions with SE aged ≥ 18 years in Texas during 2016 to 2022. Interrupted time series analyses using segmented hierarchical logistic models were fit to estimate trends in hospital mortality during the prepandemic and COVID-19 pandemic periods overall and within age, sex, and race and ethnicity strata, expressed as average marginal effects (AME). Separate hierarchical models were fit to forecast hospital mortality during the pandemic period had the pandemic not occurred (counterfactual). There were 27,885 ICU admissions with SE during the study period (33.1% aged ≥ 65 years, 58.8% racial and ethnic minority, 2.4% with COVID-19, 51.8% mechanically ventilated). Overall hospital mortality was 10.6%. On interrupted time series analyses, hospital mortality decreased during the prepandemic period overall (AME −0.31%/quarter [95% confidence interval −0.39 to −0.23]) and within all demographic strata, except those aged 18 to 44 years, whose hospital mortality was unchanged. During the pandemic period hospital mortality remained unchanged over time overall (AME −0.03%/quarter [95% confidence interval −0.18 to 0.11]) and for all demographic strata, and was consistently higher than counterfactual estimates, including following the exclusion of ICU admissions with COVID-19. Hospital mortality has decreased among critically ill patients with SE prior to the pandemic, except among younger adults. However, these outcome gains were stalled by the COVID-19 pandemic, with increased hospital deaths in this population. Determination of the specific contributions of the disruption in neurological and neuro-critical care support systems and of general health system factors to the adverse outcomes of critically ill patients with SE is paramount for future mitigation and recovery efforts. These data can inform effective implementation of care protocols in SE under population-wide health crises, as well as health policy and resource allocation. Finally, as the pandemic is receding, it is crucial to determine whether there is corresponding recovery of the prepandemic mortality trends in this population.

## 1. Introduction

Status epilepticus (SE) is a prolonged seizure state, where a single seizure failed to terminate spontaneously, leading to a prolonged seizure activity, or when repetitive seizures occur without a full return to baseline.^[1]^ It is a common neurological emergency and is associated with substantial morbidity and with high short-term mortality around 10% to 15%,^[1]^ with outcomes considered to be driven predominantly by its etiology.^[2]^ Crucially, it also has one of the most modifiable early trajectories, when timely diagnosis and interventions are applied. However, despite the substantial advances in its diagnosis and management,^[3]^ the mortality of adults with SE has not improved over the past 3 decades, according to a recent meta-analysis.^[1]^ Notably, although outcome disparities in SE were documented in cross-sectional studies across age,^[4,5]^ sex,^[4]^ and race and ethnicity^[6]^ groups, it is unknown how the outcomes of patients within these strata fared over time.

Patients with SE often require admission to intensive care unit (ICU)^[7,8]^ to facilitate seizure control, as well as for management of its predisposing conditions, with many requiring organ support, and recent studies have shown a rising demand for critical care services among these patients.^[9,10]^ Thus, the critically ill subset is likely a key driver of mortality among hospitalized patients with SE. However, evidence on the longitudinal mortality trajectories of adult critically ill patients with SE is scarce, relatively dated and, as a result, with no reports, to our knowledge, on the prognostic impact of the coronavirus disease 2019 (COVID-19) pandemic on the contemporary mortality trends in this population overall and within demographic strata.

In the abovementioned meta-analysis, there has not been a change in mortality of the subset of critically ill patients with SE.^[1]^ However, the included studies were generally small cohorts, cross-sectional, and represented patient data during the years 2003 to 2013.^[1]^ There have been, to our knowledge, only 2 longitudinal cohort studies of mortality outcomes of critically ill patients with SE. Contrasting the findings of the above-noted meta-analysis,^[1]^ decreasing hospital mortality was reported among ICU admissions with SE during 2000 to 2013 in a cohort study from Australia and New Zealand.^[11]^ In another registry-based study from the United Kingdom, hospital mortality has declined over time among ICU admissions with SE during 2001 to 2016.^[10]^ However, the generalizability of the findings of these 2 studies to other geographical regions and different health care systems is unknown, and it is unclear whether the favorable mortality trends reported in these studies continued in more recent years. Furthermore, it is unclear whether the latter outcome gains were shared equitably within individual age, sex, and race and ethnicity groups.

Finally, while the transformative impact of the COVID-19 pandemic on populations’ behavior,^[12]^ with widespread health care system strain,^[13,14]^ and with associated increase in mortality of non-COVID-19 patients with time-sensitive conditions^[15]^ and among non-COVID-19 critically ill patients in the general population,^[16]^ was well documented, the corresponding pandemic effects on the outcomes of critically ill patients with SE have not been explored. In a registry-based study from Germany, hospital mortality of patients with SE was about 2-fold higher during the pandemic compared to the prepandemic period (8.2% vs 3.8%, respectively), though the difference was not statistically significant due to the small size of the cohort (n = 328) and the characteristics and outcomes of the critically ill patients were not reported.^[17]^ Thus, the longitudinal impact of the pandemic on the contemporary mortality trajectories of critically ill patients with SE remains unknown.

A better understanding of the contemporary mortality trends of critically ill patients with SE and the impact of the COVID-19 pandemic on these trends can inform clinical care, resource allocation to mitigate the impact of future widespread health crises, and performance improvement efforts. In this study, we sought to provide a contemporary assessment of the population-level trends in hospital mortality among adult ICU patients with SE before and during the COVID-19 pandemic overall and within patients’ age, sex, and race and ethnicity strata.

## 2. Materials and methods

### 2.1. Study design and data sources

This was a retrospective, population-based cohort study. Because we used a publicly available, de-identified dataset, the study was determined to be exempt from formal review by the Texas Tech University Health Sciences Center’s Institutional Review Board. The reporting of the study findings follows the STROBE guidelines on reporting observational studies in epidemiology.^[18]^

We used the Texas Inpatient Public Use Data File (TIPUDF) to identify the target population. In brief, the TIPUDF is an administrative dataset maintained by the Texas Department of State Health Services^[19]^ and includes inpatient discharge data from state-licensed, nonfederal hospitals, and captures approximately 97% of all hospital discharges in the state.

### 2.2. Study population and outcome

Our cohort consisted of all hospitalizations aged 18 years or older with a diagnosis of SE who were admitted to ICU in acute care hospitals during 2016 to 2022. We selected 2016 as the start year for the study because the transition from International Classification of Diseases, Ninth Revision, Clinical Modification (ICD-9) to International Classification of Diseases, Tenth Revision, Clinical Modification (ICD-10) in the United States in October 2015 was found to be associated with substantial discontinuity in the frequency of identified SE hospitalizations,^[20]^ which could represent differences in the traits of captured populations and thus would confound interpretation of mortality trend analyses across the transition period. We have excluded hospitalizations without data on hospital disposition (n = 1) and hospitalizations that were transferred from or to another acute care hospital (n = 8915) because their hospital course could not be fully captured.^[8]^ We identified hospitalizations with SE as principal or secondary diagnoses,^[8,21,22]^ based on previously reported ICD-10 code taxonomy^[8]^ (Table S1, Supplemental Digital Content, https://links.lww.com/MD/O695). Hospitalizations with ICU admission were identified based on unit-specific revenue codes for an ICU or a coronary care unit. The TIPUDF dataset does not include information on the timing of ICU admission during the hospital course or patients’ clinical condition at the time of ICU admission. The primary outcome was hospital mortality among hospitalizations with SE admitted to ICU.

### 2.3. Risk-adjustment covariates

We abstracted variables selected a priori based on biological and clinical plausibility and existing literature.^[3–5,23–28]^ These included age, sex, race, and ethnicity (reported in mutually exclusive groups: non-Hispanic Black [hereafter Black], non-Hispanic White [hereafter White], Hispanic, and other), health insurance, comorbid conditions (based on the Deyo modification of the Charlson Comorbidity Index),^[29,30]^ COVID-19 (ICD-10 code U071), a do-not-resuscitate status (ICD-10 code Z66), palliative care (ICD-10 code Z515), and hospitals’ teaching status. Severity of illness was characterized using ICD-10 codes for organ dysfunction.^[31,32]^ Procedure use was identified using ICD-10 procedure codes for mechanical ventilation (5A1935Z, 5A1945Z, and 5A1955Z) and hemodialysis (5A1D00Z, 5A1D60Z, 5A1D70Z, 5A1D80Z, and 5A1D90Z). In addition, we abstracted the year and quarter of hospital admission. A quarter represents the shortest time period reported in the TIPUDF.

Because data on the etiology of SE are not available in the TIPUDF dataset, we used the approach reported by Guterman et al^[8]^ to infer the source of SE in our cohort by examining discharge diagnoses codes to abstract common causes of SE, including stroke (ischemic and hemorrhagic), central nervous system tumor, traumatic brain injury, meningoencephalitis, hyponatremia, and alcohol withdrawal. Stroke was identified by the ICD-10 codes included in Clinical Classification Software Refined (CCSR)^[33]^ categories CIR020 and CIR021; traumatic brain injury by CCSR category INJ008; central nervous system tumor by ICD-10 codes C71x, C7931, and D496; meningoencephalitis by CCSR categories NVS001 and NVS002; hyponatremia by ICD-10 code E871; and alcohol withdrawal by ICD-10 codes F10230, F10232, and F10239.

### 2.4. Statistical analysis

We summarized categorical variables as frequencies and percentages, while continuous variables were reported as mean (standard deviation). The chi-square test was used for group comparison involving categorical variables, while *t* test was used for comparison of continuous variables.

Because the TIPUDF provides discharge-level, rather than patient-level information, precluding accounting for repeated admissions, we report the number of hospitalizations and ICU admissions as units of analysis, rather than a number of patients.

We conducted interrupted time series analyses (ITSA) as our primary modeling approach to estimate trends of hospital mortality among ICU admissions with SE before and during the COVID-19 pandemic. We fit segmented hierarchical mixed-effects logistic regression models, with individual hospitals modeled as random intercepts to account for the clustering of hospitalizations within hospitals. The model included a binary intervention term equal to 0 during the prepandemic period and 1 during the COVID-19 pandemic period. The quarter of hospitalization was included as a continuous time variable over the 28 prepandemic and pandemic quarters to model prepandemic and pandemic trends. The model was adjusted for all the covariates listed in the risk-adjustment section. Multicollinearity was excluded by examination of variance inflation factors. We estimated the prepandemic (January 1, 2016–March 31, 2020) trend, level (intercept) change, pandemic (April 1, 2020–December 31, 2022)^[34–36]^ trend, and change of trends. Accordingly, the prepandemic trend is the change in hospital mortality per quarter over the 17 quarters before the pandemic; the level change represents the immediate change in hospital mortality at the start of the pandemic; the pandemic trend assessed the longitudinal impact of the pandemic on changes in hospital mortality per quarter over its 11 quarters; and change in trends is the difference in the trends of hospital mortality during the pandemic compared to the trend before the pandemic. We report model findings as average marginal effects (AME), which represent the average change in the absolute probability of hospital mortality and its 95% confidence interval (CI).

The State of Texas suppresses sex data of hospitalizations with a diagnosis of infection with the human immunodeficiency virus, and of those with alcohol or drug abuse to protect patients’ identity. Sex data were missing nonrandomly in 22.7% of hospitalizations in our cohort, precluding imputation of missing values. We used an indicator variable to model sex data for hospitalizations with suppressed sex information.

Separate ITSA models were fit to estimate trends of hospital mortality among ICU admissions with SE before and during the COVID-19 pandemic within age, sex, and race and ethnicity strata, excluding age, sex, and race and ethnicity groups from these models, respectively. Because the small number of hospital deaths in the race group “others” precluded valid trend analyses, we included only Black, Hispanic, and White ICU admissions in the trend analysis of race and ethnicity groups.

Because changes in hospital mortality among ICU admissions with SE during the pandemic period could reflect only higher mortality among those with COVID-19, rather than change in mortality trends among non-COVID-19 hospitalizations, we performed sensitivity analysis restricted to those without COVID-19.

In addition, fitting hierarchical, mixed-effects logistic models for the whole cohort, as well as for the age, sex, race and ethnicity strata, and following the exclusion of COVID-19 hospitalizations, we used the prepandemic period data to forecast time trends in hospital mortality among ICU admissions with SE during the period of April 1, 2020 to December 31, 2022, that is representing the hypothetical scenario in which the COVID-19 pandemic has not occurred (e.g., the counterfactual).^[37]^ Counterfactual values were generated by projecting values with the binary indicator of the pandemic period as 0 rather than 1.

To further quantify the impact of the COVID-19 pandemic on hospital mortality among ICU admissions with SE, we estimated the difference between the cumulative risk-adjusted probability of hospital mortality during the pandemic period based on the primary ITSA models (hereafter, termed actual) and the cumulative risk-adjusted probability of hospital mortality based on the forecasted data for the pandemic period (hereafter, termed counterfactual). These risk-adjusted probabilities of hospital mortality were derived using empirical Bayesian posterior estimates based on the hierarchal logistic models for the whole cohort and for each of the age, sex, race and ethnicity, and sensitivity analysis strata detailed above. Risk-adjusted hospital mortality data are expressed as percentages. The differences between the actual and counterfactual cumulative risk-adjusted hospital mortality are expressed as both absolute and relative values. The dependent sample *t* test was used to estimate the absolute differences between the actual and counterfactual cumulative risk-adjusted hospital mortality. Large sample two-sided confidence intervals for the population means were calculated using the pivotal method and a confidence level of 0.95. Corresponding *P* values were determined using *Z* tests.

Data management was performed using Microsoft Excel (Microsoft, Redmond, Washington) and statistical analyses were performed with R 4.0.5 (R Foundation for Statistical Computing, Vienna, Austria). A two-sided *P* value < .05 was considered statistically significant.

## 3. Results

### 3.1. Cohort characteristics

There were 27,885 ICU admissions with SE during the study period, of which 16,245 (58.3%) were hospitalized during the prepandemic period and 11,640 (41.7%) during the pandemic period.

The characteristics of ICU admissions with SE and their hospital disposition for the whole cohort and for the prepandemic and the pandemic periods are shown in Table 1. For the whole cohort, about one third (9227 [33.1%]) of ICU admissions with SE were aged ≥ 65 years, 11,287 (52.3%) were female, 16,409 (58.8%) were racial or ethnic minority, and 668 (2.4%) had COVID-19. Among all ICU admissions in our cohort, 8904 (31.9%) were classified as having a suspected source of SE, with the most common being hyponatremia (3530 [12.7%]), stroke (3221 [11.6%]), and central nervous system tumor (1230 [4.6%]).

**Table 1:** The characteristics and outcomes of ICU admissions with status epilepticus.

Variables	All*	Prepandemic*^,^ †	Pandemic*^,^ †	*P* value
	(n = 27,885)	(n = 16,245)	(n = 11,640)	
Age (years)				.219
18–44	8994 (32.3)	5198 (32.1)	3796 (32.6)	
45–64	9664 (34.7)	5605 (34.5)	4059 (34.8)	
≥65	9227 (33.1)	5442 (33.5)	3785 (32.5)	
Sex‡				.072
Female	11,287 (52.3)	6678 (52.8)	4609 (51.6)	
Male	10,274 (47.7)	5959 (47.2)	4315 (48.3)	
Race/ethnicity				.546
Black	6178 (22.2)	3625 (22.3)	2553 (21.9)	
Hispanic	8031 (28.8)	4630 (28.5)	3401 (29.2)	
White	11,476 (41.2)	6691 (41.2)	4785 (41.1)	
Other	2196 (7.8)	1295 (8.0)	901 (7.7)	
Missing	4 (<0.1)	3 (<0.1)	1 (<0.1)	
Health insurance				.464
Private	9589 (34.4)	5621 (34.6)	3968 (34.1)	
Medicare	9030 (32.4)	5198 (32.0)	3832 (32.9)	
Medicaid	4369 (15.7)	2504 (15.4)	1865 (16.0)	
Uninsured	4313 (15.5)	2493 (15.3)	1821 (15.6)	
Other	518 (1.9)	292 (1.8)	226 (1.9)	
Missing	65 (0.2)	26 (0.2)	39 (0.3)	
Deyo comorbidity index, mean (SD)	1.7 (2.2)	1.7 (2.2)	1.7 (2.1)	.394
Selected comorbidities				
Congestive heart failure	4389 (15.7)	2547 (15.7)	1842 (15.8)	.821
Chronic lung disease	4068 (14.6)	2339 (14.4)	1729 (14.8)	.705
Renal disease	4673 (16.8)	2758 (17.0)	1915 (16.5)	.271
Diabetes	8239 (29.5)	4761 (29.3)	3478 (29.9)	.278
Liver disease	1435 (5.1)	798 (4.9)	637 (5.4)	.065
Malignancy	2167 (7.8)	1258 (7.7)	909 (7.8)	.758
HIV	269 (0.9)	168 (1.0)	101 (0.9)	.398
Alcohol abuse	3285 (11.8)	1896 (11.7)	1389 (11.9)	.609
Drug abuse	5933 (21.3)	3469 (21.4)	2464 (21.2)	.687
Seizure etiologic factors				
Any suspected cause	8904 (31.9)	5166 (31.8)	3738 (32.1)	.592
Stroke	3271 (11.6)	1868 (11.5)	1403 (12.1)	.125
CNS tumor	1290 (4.6)	722 (4.4)	568 (4.8)	.115
Traumatic brain injury	795 (2.9)	455 (2.8)	340 (2.9)	.620
Meningoencephalitis	1079 (3.9)	633 (3.9)	446 (3.8)	.669
Hyponatremia	3530 (12.7)	2028 (12.5)	1502 (12.9)	.322
Alcohol withdrawal	784 (2.8)	439 (2.7)	345 (3.0)	.136
Number of organ dysfunctions, mean (SD)	1.9 (1.4)	1.8 (1.4)	1.9 (1.4)	<.001
COVID-19	668 (2.4)	NA	668 (5.7)	NA
Mechanical ventilation	14,386 (51.8)	9322 (57.4)	5064 (43.5)	<.001
Hemodialysis	1468 (5.3)	967 (6.0)	501 (4.3)	<.001
Palliative care	3424 (12.3)	1887 (11.6)	1537 (13.2)	<.001
Do-not-resuscitate status	4682 (16.8)	2688 (16.5)	1994 (17.1)	.185
Hospital disposition				
In-hospital mortality	2960 (10.6)	1758 (10.8)	1202 (10.3)	.181
Home	14,428 (51.7)	8439 (51.9)	5989 (51.5)	.788
Post-acute care facility§	7076 (25.4)	3983 (24.5)	3093 (26.6)	<.001
Hospice	2276 (8.2)	1421 (8.7)	855 (7.3)	<.001
Leave against medical advice	1145 (4.1)	644 (4.0)	501 (4.3)	.214

COVID-19 = coronavirus disease 2019, ICU = intensive care unit, HIV = human immunodeficiency virus, NA = not applicable, SD = standard deviation.

* The parenthesized figures represent percents, except for Deyo comorbidity index and number of organ dysfunctions; percentage figures may not add to 100 due to rounding.

† Prepandemic period: January 1, 2016 to March 31, 2020; pandemic period: April 1, 2020 to December 31, 2022.

‡ Sex was reported for 21,561 ICU admissions, including 12,637 during the prepandemic period and 8924 during the pandemic period; the percent figures for sex refer to the available sex data as the denominator.

§ Post-acute care facilities include: long-term acute care hospitals, inpatient rehabilitation, skilled nursing facilities, and nursing homes.

The characteristics of ICU admissions with SE during the prepandemic and pandemic periods were generally comparable. However, compared to the prepandemic period, the mean (standard deviation) number of organ dysfunctions was higher during the pandemic period (1.8 [1.4] vs 1.9 [1.4], respectively; *P* < .001), as was the use of palliative care (11.6% vs 13.2%, respectively; *P* < .001), while the use of mechanical ventilation and hemodialysis was lower (57.4% vs 43.5% and 6.0% vs 4.3%, respectively; *P* < .001 for both).

### 3.2. Cohort outcomes

Among ICU admissions with SE, the aggregate rates of hospital mortality and home discharge were similar between the prepandemic and pandemic periods (10.8% vs 10.3% [*P* = .181] and 51.9% vs 51.5% [*P* = .788, respectively]). On the other hand, compared to the prepandemic period, the rates of discharge to post-acute care facilities rose during the pandemic period, while the rate of discharge to hospice decreased (24.5% vs 26.6% and 8.7% vs 7.3%, respectively; *P* < .001 for both).

### 3.3. Trends in hospital mortality before and during the COVID-19 pandemic

Comparisons of crude hospital mortality between the first and last quarters of prepandemic period and between the first and last quarters of the pandemic period for the whole cohort and within examined strata are detailed in Table 2. Hospital mortality for the whole cohort has decreased between the first and last quarters of the prepandemic period from 14.9% to 9.1% (*P* < .001), respectively, while remaining unchanged during the pandemic (10.6% vs 10.1%, respectively; *P* = .707). Similar changes were generally observed within the examined strata.

**Table 2:** Hospital mortality over the pre-pandemic and COVID-19 pandemic periods among ICU admissions with status epilepticus.

Group	Combined, 2016–2022*	Pre-pandemic period*^,^ †		*P* valueǁ	Pandemic period*^,^ ‡		*P* valueǁ
		Q1§ 2016	Q1§ 2020		Q2§ 2020	Q4§ 2022	
All	2960/27,885 (10.6)	129/864 (14.9)	104/1141 (9.1)	<.001	102/960 (10.6)	118/1164 (10.1)	.707
All, excluding COVID-19	2819/27,217 (10.4)	129/864 (14.9)	104/1141 (9.1)	<.001	96/942 (10.2)	111/1121 (9.9)	.821
Age (years)							
18–44	466/8994 (5.2)	10/261 (3.8)	14/358 (3.9)	.949	22/290 (7.6)	20/401 (5.0)	.159
45–64	1078/9664 (11.2)	46/321 (14.3)	34/387 (8.8)	.021	40/332 (12.0)	52/367 (14.2)	.390
≥65	1416/9227 (15.3)	73/281 (25.9)	56/396 (14.1)	<.001	40/338 (11.8)	46/396 (11.6)	.933
Sex							
Female	1286/11,287 (11.4)	66/385 (17.1)	44/487 (9.0)	<.001	46/402 (11.4)	47/455 (10.3)	.605
Male	1209/10,274 (11.8)	48/309 (15.5)	44/393 (11.1)	.086	36/332 (10.8)	56/472 (11.9)	.629
Race and ethnicity							
Black	572/6178 (9.3)	27/166 (16.3)	23/300 (7.7)	.042	20/219 (9.1)	28/256 (10.9)	.516
Hispanic	839/8031 (10.4)	34/267 (12.7)	22/291 (7.6)	.045	26/231 (11.2)	40/363 (11.0)	.939
White	1308/11,476 (11.4)	61/356 (17.1)	48/466 (10.3)	.004	51/441 (11.6)	40/441 (9.1)	.725

COVID-19 = coronavirus disease 2019, ICU = intensive care unit.

* Parenthesized figures represent percentages.

† Pre-pandemic period: January 1, 2016 to March 31, 2020.

‡ Pandemic period: April 1, 2020 to Decemeber 31, 2022.

§ Q1, Q2, Q4: quarters of a specific calendar year.

ǁ The *P* values compare hospital mortality between the first and last quarters of the prepandemic period and between the first and last quarters of the pandemic period.

**Figure 1.**
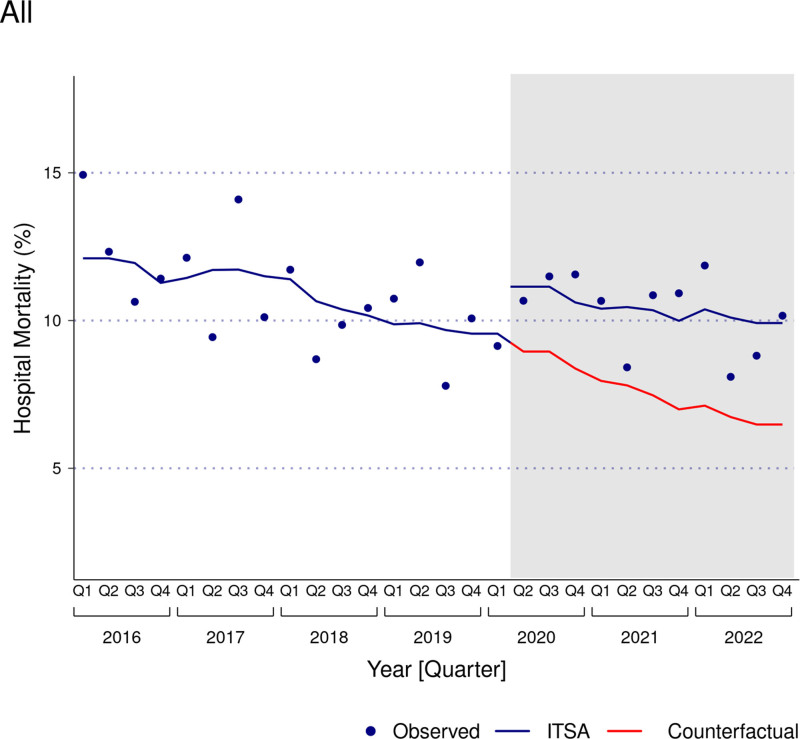
Interrupted time series analysis of temporal trends in hospital mortality of all ICU admissions with status epilepticus, 2016 to 2022. The shaded area represents the COVID-19 pandemic period. The dots represent the observed hospital mortality for each quarter. The blue-colored line represents the temporal trend of risk-adjusted hospital mortality, derived from segmented hierarchical mixed-effects logistic regression. The red-colored line represents the counterfactual trend of hospital mortality among ICU admissions, had the COVID-19 pandemic not occurred. The regression model was adjusted for age, sex, race and ethnicity, health insurance, Deyo comorbidity index, suspected etiology of status epilepticus, COVID-19, number of organ dysfunctions, mechanical ventilation, hemodialysis, do-not-resuscitate status, palliative care, and hospitals’ teaching designation. COVID-19 = coronavirus disease 2019, ICU = intensive care unit, ITSA = interrupted time series analysis.

**Figure 2.**
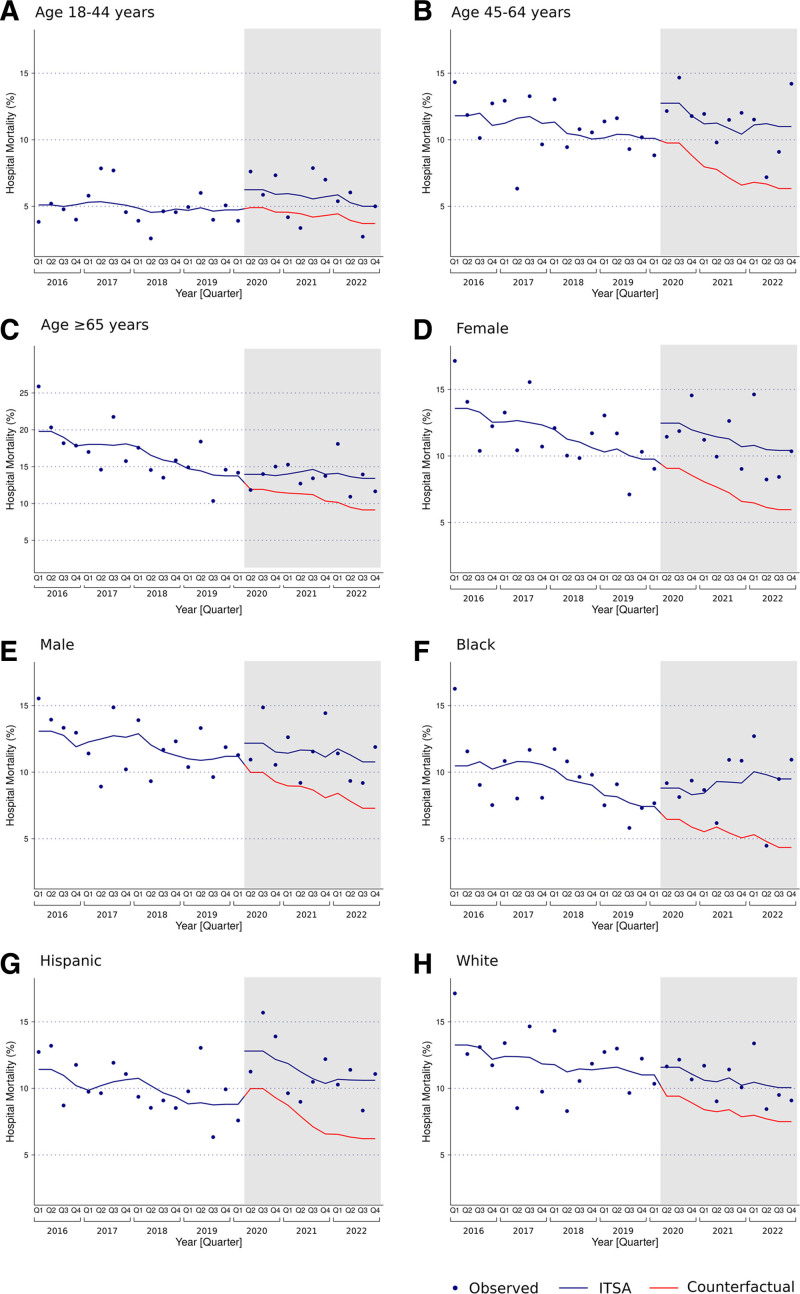
Interrupted time series analysis of temporal trends in hospital mortality of ICU admissions with status epilepticus stratified by age, sex, and race and ethnicity, 2016 to 2022. The shaded area represents the COVID-19 pandemic period. The dots represent the observed hospital mortality for each quarter. The blue-colored lines represent temporal trends of risk-adjusted hospital mortality, derived from segmented hierarchical mixed-effects logistic regression. The red-colored lines represent counterfactual trends of hospital mortality among ICU admissions, had the COVID-19 pandemic not occurred. The regression model was adjusted for age, sex, race and ethnicity, health insurance, Deyo comorbidity index, suspected etiology of status epilepticus, COVID-19, number of organ dysfunctions, mechanical ventilation, hemodialysis, do-not-resuscitate status, palliative care, and hospitals’ teaching designation. Age, sex, race, and ethnicity were excluded from the models of mortality trends within age, sex, and race and ethnicity groups, respectively. COVID-19 = coronavirus disease 2019, ICU = intensive care unit, ITSA = interrupted time series analysis.

Risk-adjusted trends of hospital mortality among ICU admissions with SE before and during the COVID-19 pandemic for the whole cohort and within demographic groups are detailed in Table 3, Figures 1 and 2, and in Figure S1, Supplemental Digital Content, https://links.lww.com/MD/O696. During the prepandemic period, hospital mortality decreased for the whole cohort (AME −0.31%/quarter [95% CI −0.39 to −0.23]) and within each of the examined age, sex, and race and ethnicity strata, except among those aged 18 to 44 years, where hospital mortality did not change over time (AME −0.09%/quarter [95% CI −0.19 to 0.01]). Hospital mortality remained unchanged during the pandemic period for the whole cohort (AME −0.03%/quarter [95% CI −0.18 to 0.11]) and within the age, sex, and race and ethnicity groups.

**Table 3:** Interrupted time series of trends in hospital mortality of ICU admissions with status epilepticus before and during the coronavirus disease 2019 pandemic.

Group	Pre-COVID-19 pandemic	*P* value	Level change associated with COVID-19	*P* value	During COVID-19 pandemic	*P* value	Trend change associated with the	*P* value
	(January 2016–March 2020)		pandemic (April–June 2020)†		(April 2020–December 2022)		COVID-19 pandemic‡	
	Time trend (95% CI)*		Level change (95% CI)*		Time trend (95% CI)*		Trend change (95% CI)^a^	
All	-0.31 (-0.39 to -0.23)	<.001	1.65 (0.39 to 2.92)	.010	-0.03 (-0.18 to 0.11)	.646	0.27 (0.11 to 0.44)	.001
All, excluding COVID-19	-0.30 (-0.38 to -0.22)	<.001	1.29 (0.02 to 2.56)	.046	0.02 (-0.13 to 0.17)	.797	0.32 (0.15 to 0.49)	<.001
Age (years)								
18–44	-0.09 (-0.19 to 0.01)	.070	1.20 (-0.29 to 2.69)	.114	-0.05 (-0.22 to 0.12)	.541	0.04 (0.16 to 0.23)	.706
45–64	-0.34 (-0.47 to -0.21)	<.001	2.19 (0.07 to 4.72)	.043	0.03 (-0.21 to 0.27)	.795	0.37 (0.10 to 0.65)	.008
≥65	-0.49 (-0.66 to -0.33)	<.001	1.13 (-1.58 to 3.84)	.412	-0.04 (-0.36 to 0.28)	.803	0.45 (0.09 to 0.81)	.0145
Sex								
Female	-0.38 (-0.51 to -0.25)	<.001	2.85 (0.73 to 4.96)	.008	-0.07 (-0.31 to 0.17)	.564	0.31 (0.03 to 0.59)	.0277
Male	-0.33 (-0.46 to -0.19)	<.001	1.61 (-0.56 to 3.77)	.146	-0.04 (-0.29 to 0.21)	.736	0.29 (0.01 to 0.57)	.049
Race and ethnicity								
Black	-0.36 (-0.52 to -0.20)	<.001	1.29 (-1.25 to 3.83)	.320	0.21 (-0.08 to 0.49)	.162	0.57 (0.23 to 0.90)	<.001
Hispanic	-0.28 (-0.43 to -0.13)	.001	1.86 (-0.56 to 4.27)	.132	0.07 (-0.19 to 0.34)	.587	0.35 (0.05 to 0.66)	.024
White	-0.33 (-0.45 to -0.20)	<.001	2.07 (0.09 to 4.04)	.040	-0.22 (-0.45 to 0.02)	.068	0.11 (-0.16 to 0.38)	.416

95% CI = 95% confidence interval, COVID-19 = coronavirus disease 2019, ICU = intensive care unit.

* Coefficients represent average marginal effects (AME), representing absolute change in the probability of hospital mortality (for instance: AME 1.5 indicates increase in-hospital mortality by 1.5% [e.g., from 10.0% to 11.5%]). AME for time trends (pre-pandemic and during the pandemic period) are expressed per quarter.

† Level change represents the immediate change in hospital mortality at the start of the COVID-19 pandemic.

‡ Change in trends represents change in pandemic versus pre-pandemic hospital mortality time trends.

The actual and counterfactual cumulative risk-adjusted hospital mortality estimates among ICU admissions with SE for the whole cohort and for the demographic categories are presented in Table 4. During the COVID-19 pandemic, the actual and counterfactual cumulative risk-adjusted hospital mortality among ICU admissions with SE was 10.3% (95% CI 10.0–10.7) and 7.5% (95% CI 7.2–7.8), respectively, representing a 38.1% (95% CI 37.9–38.3) increase. Actual cumulative risk-adjusted hospital mortality among ICU admissions with SE during the pandemic period was also higher than the counterfactual mortality estimates within each of the examined age, sex, and race and ethnicity categories.

**Table 4:** Th difference between the actual and counterfactual cumulative risk-adjusted hospital mortality during the coronavirus disease 2019 pandemic period among ICU admissions with status epilepticus.

Group	Risk-adjusted hospital mortality		Absolute difference (95% CI)†,§, ¶
	Actual (95% CI)*,†	Counterfactual (95% CI)†,‡	
All	10.3 (10.0–10.7)	7.5 (7.2–7.8)	2.8 (2.7–2.9)
All, excluding COVID-19ǁ	9.7 (9.4–10.0)	7.0 (6.7–7.3)	2.7 (2.6–2.8)
Age (years)			
18–44	5.1 (4.7–5.4)	3.5 (3.2–3.8)	1.6 (1.5–1.7)
45–64	11.1 (10.5–11.8)	8.2 (7.7–8.7)	2.9 (2.8–3.0)
≥65	14.6 (14.0–15.3)	10.7 (10.2–11.2)	3.9 (3.8–4.1)
Sex			
Female	11.0 (10.5–11.5)	8.0 (7.5–8.4)	3.0 (2.9–3.1)
Male	11.6 (11.0–12.2)	8.5 (8.0–9.0)	3.1 (3.0–3.2)
Race and ethnicity			
Black	9.0 (8.4–9.6)	6..5 (6.0–7.0)	2.5 (2.4–2.7)
Hispanic	10.3 (9.7–10.9)	7.4 (6.9–7.9)	2.9 (2.7–3.0)
White	11.1 (10.6–11.6)	8.1 (7.7–8.6)	3.0 (2.9–3.1)

CI = confidence interval, COVID-19 = coronavirus disease 2019, ICU = intensive care unit.

* Actual cumulative risk-adjusted hospital mortality during the pandemic period (April 1, 2020–December 31, 2022).

† Mortality data and differences between actual and counterfactual estimates are expressed as percentages.

‡ Counterfactual cumulative risk-adjusted hospital mortality for the pandemic period (April 1, 2020–December 31, 2022), forecasted based on prepandemic (January 1, 2020–March 31, 2020) trends.

§ Absolute difference between the actual and counterfactual cumulative risk-adjusted hospital mortality during the pandemic period.

ǁ The cohort of ICU admissions with status epilepticus without those with a diagnosis of COVID-19.

¶ *P* < .001 for all comparisons.

The findings on sensitivity analysis, after excluding ICU admissions with SE with a diagnosis of COVID-19, were consistent with those of the primary model for the whole cohort (Tables 2 and 3, and Figure S2, Supplemental Digital Content, https://links.lww.com/MD/O697). As in the primary model, hospital mortality remained unchanged during the pandemic period (AME 0.02%/quarter [95% CI −0.13 to 0.17]). The actual cumulative risk-adjusted hospital mortality for the pandemic period among non-COVID-19 ICU admissions with SE was 38.3% (95% CI 38.1–38.5) higher than the counterfactual.

## 4. Discussion

### 4.1. Key findings

In this population-based study of adult ICU admissions with SE, hospital mortality decreased over time in the years prior to the COVID-19 pandemic overall and within age, sex, and race and ethnicity groups, except among younger adults. During the pandemic period, risk-adjusted hospital mortality was stable and higher than predicted for the whole cohort and within the examined demographic strata. Higher than predicted risk-adjusted hospital mortality among ICU admissions with SE during the pandemic period remained on analyses restricted to non-COVID-19 hospitalizations. To our knowledge, this study represents the first longitudinal examination of hospital mortality of adult critically ill patients with SE in the United States and of the impact of the COVID-19 pandemic on these outcomes in this population.

### 4.2. Relationship with prior studies

The overall hospital mortality rate for the whole cohort in our study is within the range of reported mortality among adult ICU admissions with SE in the meta-analysis by Neligan et al (10.6% vs 16.8% [95% CI 10.1–23.5],^[1]^ respectively), and is similar to that reported by Damian et al in the UK (10.5%).^[10]^ On the other hand, hospital mortality was markedly lower (4.7%) among ICU admissions with SE reported by Hay et al.^[11]^ However, the mortality rates in that study are not directly comparable to our cohort and other ICU-based studies of SE because it focused only on patients with SE as the primary diagnosis of ICU admission who did not have SE secondary to new brain pathology diagnosed on that same admission, including stroke, brain tumor, and traumatic brain injury, or cardiac arrest prior to ICU admission.^[11]^

To our knowledge, this study is the first to examine the temporal trajectories of short-term mortality trends within the demographic traits of ICU admissions with SE. Although disparities in hospital mortality were observed, predominantly within the age and race and ethnicity strata, we show that the outcome improvement for the whole cohort during the prepandemic period was shared by all groups, except among those aged 18 to 44 years. The factors underlying the unchanged hospital mortality in the latter group are unclear. There may have been differential changes in case mix over time across age groups, not captured in our data, which may have affected the outcomes of these patients differently than older ones. In addition, care of critically ill patients with SE could have improved over time, but possibly did not have significant additional beneficial prognostic effect in younger patients, who had already the lowest hospital mortality compared to older counterparts.

Our findings of decreasing mortality during the prepandemic period among ICU admissions with SE for the whole cohort are consistent with and extend to the more recent period the estimates in the other 2 longitudinal ICU-based studies of SE.^[10,11]^ However, our findings contrast the reported lack of evidence of a decrease in mortality over time in the meta-analysis by Neligan et al,^[1]^ though the factors underlying these discordant findings are unclear. The authors of the meta-analysis hypothesized that the lack of mortality improvement over time may reflect findings that treatment protocols are not followed in many patients with SE and that advances in critical care have resulted in sicker patients surviving longer and subsequently developing SE.^[1]^ In addition, it has been suggested that increased use of electroencephalography in the ICU and population aging, making SE increasingly a disease of the elderly, could have contributed to the reported lack of improvement in mortality of patients with SE.^[38,39]^ It is plausible, however, that 1 or more of these potential adverse prognostic factors was present in our cohort and those reported by Damian et al^[10]^ and Hay et al.^[11]^ Nevertheless, it was shown that in a large cohort of patients with SE, treatment adherence to recommendations appeared to play a negligible prognostic role.^[40]^ In addition, although older ICU admissions with SE in our cohort had, as expected,^[3]^ the highest hospital mortality across the examined age groups, their risk-adjusted mortality rates during the prepandemic period decreased at the fastest pace. Moreover, the decreased hospital mortality among ICU admissions with SE in the study by Damian et al took place despite the rise in the age of their patients over time.^[10]^ It may be postulated that some patient characteristics, not captured in our data, may have changed over time and contributed to decreased mortality in our cohort. In addition, as noted earlier, the decrease in prepandemic hospital mortality among ICU admissions with SE in our cohort may reflect improvements in patient management. However, our dataset precludes further inferences on either of these potential mechanisms. Further studies, using more granular data, are needed for a better understanding of the drivers of the observed mortality trends in our population, as well as the differential outcome trends across age strata.

We show that the COVID-19 pandemic has brought to a halt the progressive decline in hospital mortality in the preceding years among ICU admissions with SE in our cohort, overall and generally within the examined patients’ demographics. Thus, pandemic era actual cumulative risk-adjusted hospital mortality among these patients was 38.1% higher than the counterfactual for that period for the whole cohort and the quarterly risk-adjusted hospital mortality remained unchanged over time during that period. Similar prognostic setbacks took place within the examined demographic strata. Notably, actual cumulative risk-adjusted hospital mortality among ICU admissions with status epileptics during the pandemic period in our cohort was higher than the counterfactual even following the exclusion of hospitalizations with COVID-19.

The higher hospital mortality among ICU admissions with SE during the pandemic may have been driven by both prehospital and hospital-level factors. The COVID-19 pandemic has been associated with a substantial increase in the severity of illness^[41]^ and, specifically, illness requiring critical care,^[42]^ among patients presenting to the Emergency Department, compared to the prepandmeic period, which could have increased patients’ risk of death. These pandemic-associated changes in baseline illness severity may have been driven by reported prevalent delays in health care related to avoidance of medical encounters due to patients’ concerns of exposure to COVID-19,^[43,44]^ but also due to increased difficulties getting an appointment,^[43,45]^ or access to care location.^[45]^ Commonly missed appointments for routine care and care for serious problems^[45]^ were often reported to have negative health consequences.^[45]^ Specifically, such care delays may have adversely affected control of chronic comorbid conditions in general,^[46]^ those predisposing to SE, and, possibly, control of acute problems readily managed in the outpatient setting. Moreover, once acute health crises have developed, requiring emergent hospital care, pandemic-related strains on Emergency Medical Services have resulted in delayed on-site care^[47]^ and in arrival to the Emergency Department.^[48]^ At the hospital level, broad pandemic-induced strains took place, including those on critical care services,^[49]^ often with associated delays in admission to ICU,^[50,51]^ and with a reported decrease in neurological services support.^[52]^ Together, these health system strains have likely affected processes of care, which could have been especially consequential for the outcomes of pandemic era patients with time-sensitive conditions,^[15]^ including those with SE admitted to ICU, contributing to the reversal of outcome gains during the prepandemic period among the latter. However, the administrative data in this study do not allow direct assessment of these potential hospital-level factors, nor include prehospital information.

### 4.3. Implications of study findings

Our findings demonstrate that despite the numerous previously reported challenges in the care of patients with SE, there has been substantial and progressive improvement in the short-term mortality of the critically ill subset in the years immediately prior to the COVID-19 pandemic. These estimates can inform future performance improvement efforts in this population.

Although broad commonalities can be drawn in future planning for the care of time-sensitive conditions in general under pandemic conditions, our findings highlight the need for a more systematic examination of population-level crisis-induced stress areas across the healthcare continuum that affect the presentation and care of patients with SE. Effective implementation of existing and future care protocols, including for SE, cannot be optimized without identifying and properly addressing condition-specific systemic factors that may result in care barriers. The quantitative estimates of the impact of the COVID-19 pandemic on the trajectories of short-term mortality of critically ill patients with SE in our study, both overall and over time, along with their postulated system-level drivers, can serve as a starting point to inform future efforts to identify and quantify the contribution of specific prehospital and hospital- and health system-level factors to the adverse outcomes observed in this population.

Specifically, using more granular data, examination of the spatial and temporal pandemic-induced strains affecting patient health-related behaviors and prehospital neurological care and in hospital-level neurological and neuro-critical care support across healthcare systems and quantifying their impact on the intersection between changes in processes of care and patient outcomes can inform future, priority-based, targeted disaster preparedness efforts for neurological emergencies, including SE, to mitigate the adverse impact of future population-wide health crises. Finally, as the current pandemic has officially receded in the United States, tracking the post-pandemic outcomes of critically ill patients with SE is crucial to determine whether there is corresponding recovery of the prepandemic mortality trends in this population.

### 4.4. Strengths and limitations

This study leverages a population-based high-quality administrative dataset for the most contemporary estimates of mortality trends of adult critically ill patients with SE, including the impact of the COVID-19 pandemic. Our cohort findings represent real-world estimates of the trajectories of short-term outcomes of unselected patients with SE who are managed in the ICU during their hospital stay.

Our study has, however, several inherent limitations, in addition to those noted earlier, related to its retrospective design and use of administrative data. First, the use of ICD codes could have led to group misclassifications. Second, although our ICD-10 code-based taxonomy for identifying hospitalizations with SE was used by other investigators,^[8]^ it has not been validated since the transition to ICD-10 coding in the United States. Third, the suppression of sex data by the State of Texas in specific patient strata has likely affected our trend estimates within the sex groups. Because alcohol and drug abuse are generally more common among males,^[53]^ and because the COVID-19 pandemic was associated with increased alcohol and drug abuse,^[54]^ the relatively close estimates of hospital mortality between females and males in our cohort may not be fully representative of their outcomes among ICU admissions with SE in general and specifically during the pandemic. Fourth, our dataset did not include information on the etiology of SE, its duration, ICU and hospital strains, and processes of care, which may have varied over time overall and across the demographic strata. Thus, we cannot exclude residual confounding in our models. Last, our prepandemic estimates of hospital mortality among ICU admissions with SE and the impact of the pandemic on patient outcomes may not be generalizable to other geographical areas and health systems.

## 5. Conclusions

Among adult ICU admissions with SE, hospital mortality has declined over time prior to the onset of the COVID-19 pandemic overall and within age, sex, and race and ethnicity groups, except among younger adults. Hospital mortality remained unchanged during the pandemic and was substantially higher than expected had the pandemic not taken place for the whole cohort, across examined demographic strata, and following the exclusion of ICU admissions with a diagnosis of COVID-19.

## Author contributions

**Conceptualization:** Lavi Oud.

**Data curation:** Lavi Oud, John Garza.

**Formal analysis:** Lavi Oud, John Garza.

**Investigation:** Lavi Oud, John Garza.

**Methodology:** Lavi Oud, John Garza.

**Project administration:** Lavi Oud.

**Resources:** Lavi Oud.

**Software:** John Garza.

**Supervision:** Lavi Oud, John Garza.

**Validation:** Lavi Oud, John Garza.

**Visualization:** Lavi Oud, John Garza.

**Writing – original draft:** Lavi Oud.

**Writing – review & editing:** Lavi Oud, John Garza.

## Supplementary Material


