# Supplementary material for: Trends in hospital mortality of patients with status epilepticus in the ICU before and during the COVID-19 pandemic
Source: Medicine (Baltimore). 2025 Apr 25;104(17):e42219. doi: 10.1097/MD.0000000000042219 (PMC12039991; doi:10.1097/MD.0000000000042219)
Supplement: Supplementary file 1 [file medi-104-e42219-s001.docx]

**Table S1. International Classification of Diseases*,* Tenth Revision*,* Clinical Modification (ICD-10-CM) codes used to identify status epilepticus.**

**ICD-10-CM codes**

G40A01, G40A11, G40B01, G40B11, G40001, G40011, G40101, G40111, G40201, G40211, G40301, G40311, G40401, G40411, G40501, G40801, G40803, G40811, G40813, G40821, G40823, G40901, G40911
