# Supplementary figures and images for: Trends in hospital mortality of patients with status epilepticus in the ICU before and during the COVID-19 pandemic
Source: Medicine (Baltimore). 2025 Apr 25;104(17):e42219. doi: 10.1097/MD.0000000000042219 (PMC12039991; doi:10.1097/MD.0000000000042219)

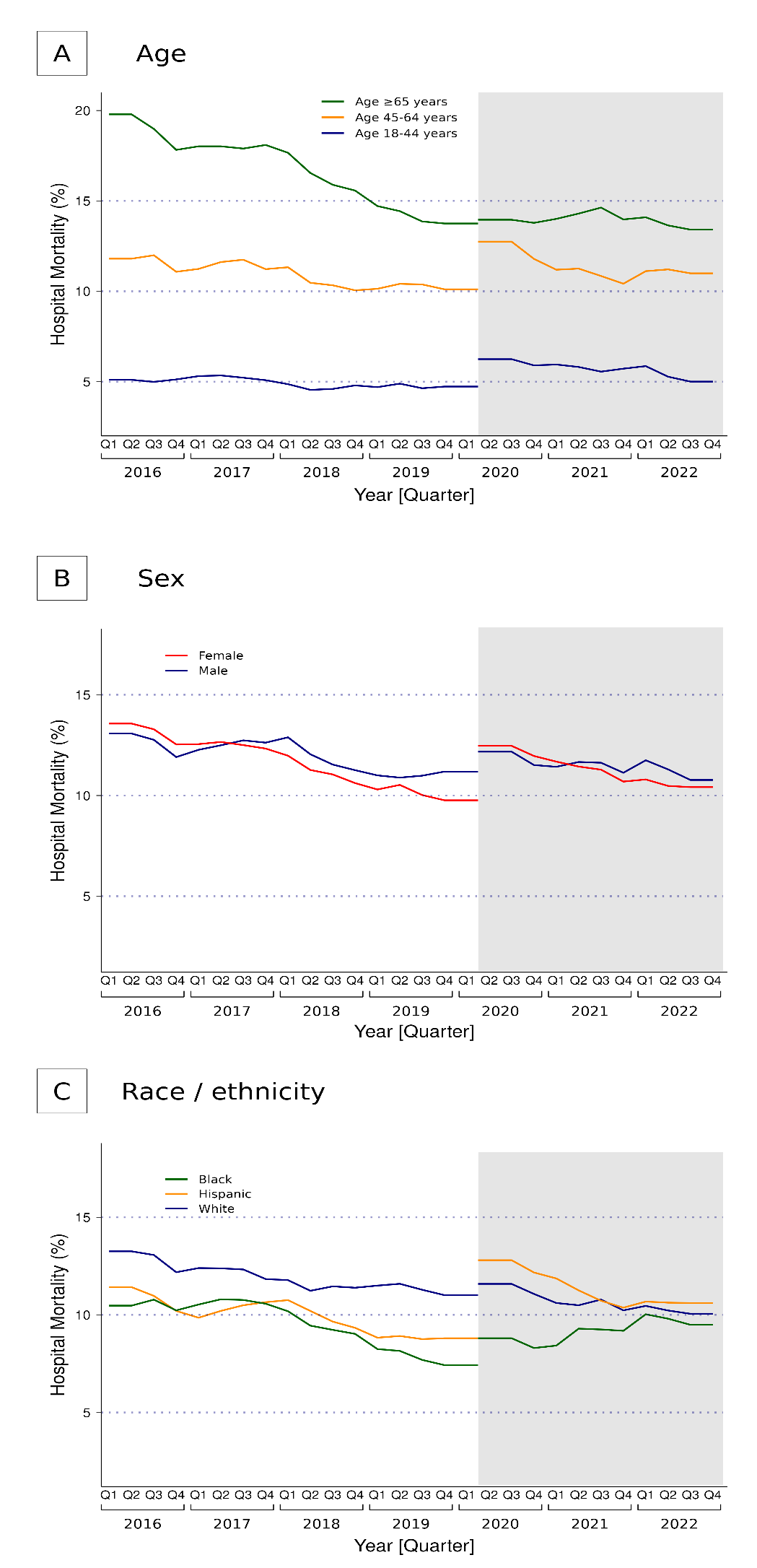

Supplement: Supplementary file 2 [file medi-104-e42219-s002.docx]

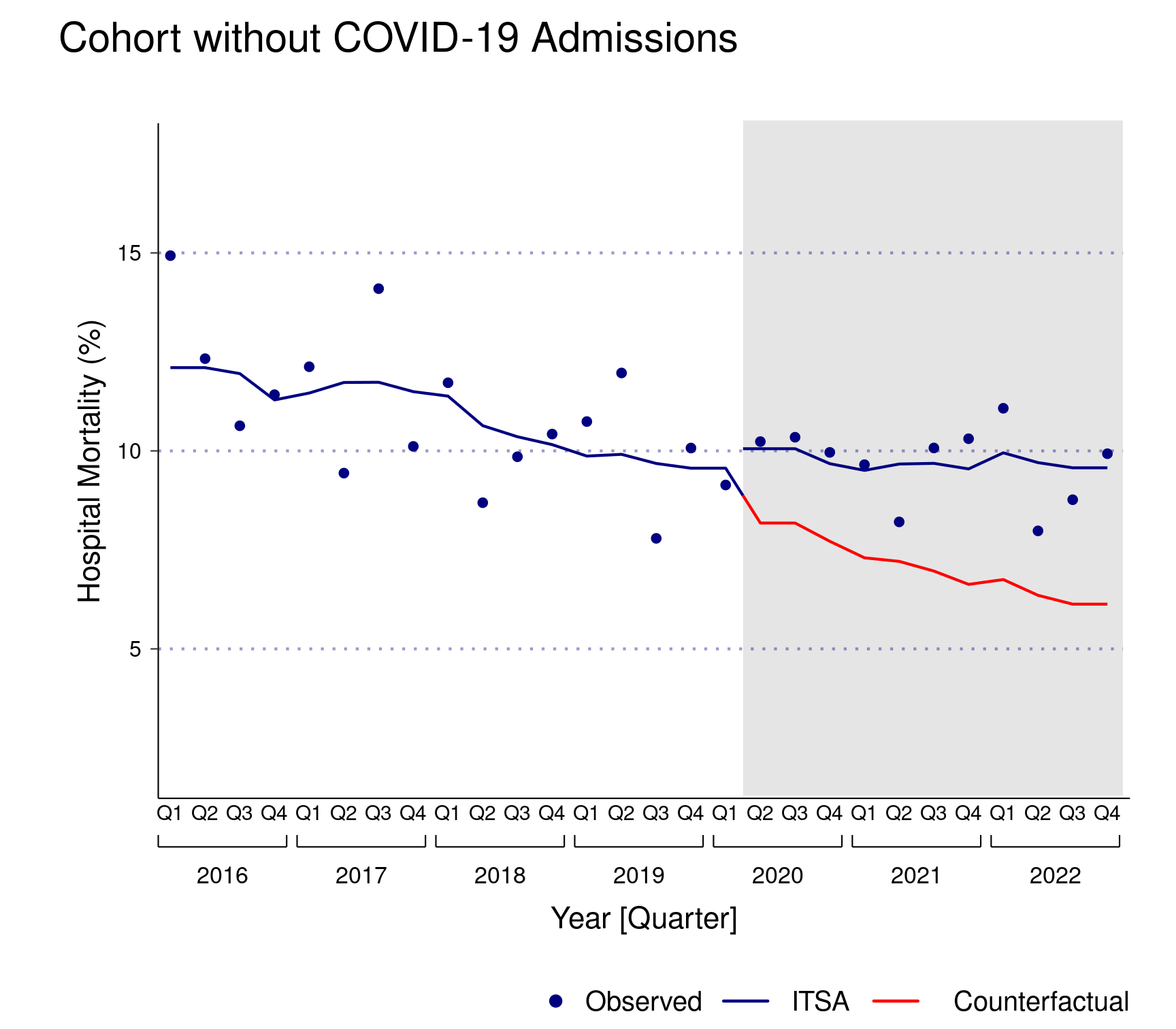

Supplement: Supplementary file 3 [file medi-104-e42219-s003.docx]
